# Body size in early life and the risk of postmenopausal breast cancer

**DOI:** 10.1186/s12885-022-09233-9

**Published:** 2022-03-08

**Authors:** TienYu Owen Yang, Benjamin J. Cairns, Kirstin Pirie, Jane Green, Valerie Beral, Sarah Floud, Gillian K. Reeves

**Affiliations:** Cancer Epidemiology Unit, Nuffield Department of Population Health, University of Oxford, Richard Doll Building, Old Road Campus, Oxford, OX3 7LF UK

**Keywords:** Adiposity, early life body size, postmenopausal breast cancer, oestrogen receptor status, breast cancer subtypes

## Abstract

**Background:**

Greater early life adiposity has been reported to reduce postmenopausal breast cancer risk but it is unclear whether this association varies by tumour characteristics. We aimed to assess associations of early life body size with postmenopausal breast cancer and its subtypes, allowing for body size at other ages.

**Methods:**

A total of 342,079 postmenopausal UK women who reported their body size at age 10, clothes size at age 20, and body mass index (BMI) at baseline (around age 60) were followed by record linkage to national databases for cancers and deaths. Cox regression yielded adjusted relative risks (RRs) of breast cancer, overall and by tumour subtype, in relation to body size at different ages.

**Results:**

During an average follow-up of 14 years, 15,506 breast cancers were diagnosed. After adjustment for 15 potential confounders, greater BMI at age 60 was associated with an increased risk of postmenopausal breast cancer (RR per 5 kg/m^2^=1.20, 95%CI 1.18-1.22) whereas greater adiposity in childhood and, to a lesser extent, early adulthood, was associated with a reduced risk (0.70, 0.66-0.74, and 0.92, 0.89-0.96, respectively). Additional adjustment for midlife BMI strengthened associations with BMI at both age 10 (0.63, 0.60-0.68) and at age 20 (0.78, 0.75-0.81). The association with midlife adiposity was confined to hormone sensitive subtypes but early life adiposity had a similar impact on the risk of all subtypes.

**Conclusion:**

Early life and midlife adiposity have opposite effects on postmenopausal breast cancer risk and the biological mechanisms underlying these associations are likely to differ.

**Supplementary Information:**

The online version contains supplementary material available at 10.1186/s12885-022-09233-9.

## Background

Adiposity has qualitatively different associations with pre- and postmenopausal breast cancer [1]. Greater adult adiposity has consistently been found to be associated with a decreased risk of breast cancer before the menopause but an increased risk after the menopause, and these associations are greater for oestrogen receptor (ER) positive disease [1]. The qualitatively different associations of BMI with risk in pre- and postmenopausal women are likely to reflect the marked difference in circulating levels of oestradiol, and other hormones, after the menopause, and the fact that adipose tissue takes over from the ovaries as the main source of oestrogen after menopause. Several prospective studies have found greater early life body size to be associated with a decreased risk of postmenopausal breast cancer [2–5] but it is unclear whether these associations differ by ER status or other tumour characteristics.

We examined associations of prospective, validated information on body size in childhood and early adulthood, and midlife adiposity, with the risk of postmenopausal breast cancer and its main subtypes, as defined by histology, grade, and ER, progesterone receptor (PR), and human epidermal growth factor receptor 2 (HER2) status, in a large study of UK women.

## Methods

### Data collection, definitions and follow-up

Between 1996 and 2001, 1.3 million women in the UK were recruited into the Million Women Study, an open ended prospective study of women in England and Scotland, when they were invited for routine screening through the NHS Breast Screening Programme. Participants completed a written questionnaire on lifestyle and other personal characteristics at recruitment, and were re-surveyed at 3-5 year intervals. The study includes around 1 in 4 UK women born in 1935-1950 with a wide range of backgrounds, behaviours and lifestyles at recruitment, and their characteristics are broadly similar to those of all UK women in the relevant age range at that time. The study website (www.millionwomenstudy.org) and cohort profile [6] provide full details of study design and data access. Participants gave written informed consent for follow-up and ethical approval was provided by Oxford and Anglia Multi-Centre Research Ethics committee.

Women were asked for the first time about early life body size at the survey in median year 2001 (IQR 2000-2003), which forms the baseline for these analyses. At that survey, women were asked if they were of average size, thinner or plumper than their peers at age 10; and about clothes size at age 20. These self-reported measures of early life body size correspond well with BMI measured at age 11 and age 20, respectively, in 541 study participants who also took part in the National Survey of Health and Development (NSHD), with Spearman correlations of 0.51 and 0.63, respectively [7].

Women first reported their current height and weight at recruitment, and were asked again about their weight at subsequent resurveys. BMI at baseline was, therefore, generally calculated using self-reported height at recruitment and self-reported weight at the baseline survey unless information on weight at baseline survey was missing, in which case weight at recruitment was used instead. As part of a previously published validation study of self-reported anthropometric variables in the Million Women Study, clinical measurements of height and weight were obtained in a subsample of ~4000 participants in median year 2008 [8]. In this subsample, measured BMI was highly correlated with BMI self-reported at a similar time point (median year 2008) (r=0.95) and with BMI self-reported at baseline survey (r=0.88).

The cohort is followed for cancer registrations, emigrations and deaths, by record linkage to routinely collected national data [6]. Cancer and cause of death are coded according to the International Classification of Diseases, 10^th^ Revision. The main endpoint of interest was incident invasive breast cancer (ICD10 C50).

Information on tumour characteristics among women diagnosed with breast cancer was derived from cancer registration data, medical records, and questionnaire data, and used to classify breast cancers according to histology, grade and joint ER, PR and HER2 status. Since information on ER status was relatively more complete in these data than that for other markers, analyses of body size in relation to ER status included all breast cancers with known ER status, regardless of whether they had information on other tumour markers. Comparisons of associations with more refined subtypes were further restricted to breast cancers with information on relevant combinations of ER, PR and HER2 status. Among women with known ER and PR status, we examined associations separately for ER+PR-, ER+PR+ and ER-PR- cancers, as ER-PR+ breast cancer is not generally considered to be a reproducible subtype [9]. Among women with information on ER and HER2 status, we also conducted analyses separately for the four categories of cancers defined as: ER+HER2-, ER+HER2+, ER-HER2+, and ER-HER2-, which were taken to represent the four intrinsic subtypes [10], subsequently referred to as luminal A, luminal B, HER2-enriched, and basal-like cancers, respectively. Although PR status can be used in addition to ER and HER2 status to provide an immunohistochemical representation of the four main molecular subtypes, it is strongly correlated with ER status, and so use of PR status in addition to ER and HER2 status would be unlikely to have yielded substantially different groupings.

### Statistical methods

The baseline for these analyses was the survey in median year 2001 (IQR 2000-2003) (average age at completion 61 years [SD 5]). Women who did not report body size at age 10, clothes size at age 20, and/or recent height and weight were excluded, as were women diagnosed before baseline with *in situ* breast cancer (ICD10 D05) or invasive cancer other than non-melanoma skin cancer (ICD10 C44). Since menopausal hormone therapy (MHT) use attenuates the association between BMI and breast cancer [1] ever users of MHT were excluded from the main analyses but were included in sensitivity analyses.

Women contributed person-years from date of completion of the baseline survey, if they reported being postmenopausal by then, or from age 55 otherwise, since most women reach menopause by this age [11]. Person-years were contributed up to date of cancer diagnosis (excluding non-melanoma skin cancers), date of death or emigration, or 31 December 2017, whichever was earliest.

Cox regression models (STATA 15.1) were used to estimate adjusted relative risks of postmenopausal breast cancer by categories of the three main exposure variables: body size at age 10 (thin, average, or plump compared to peers); clothes size at age 20 (UK size <12, 12, 14 and 16+); and BMI at baseline (<25.0, 25.0-29.9, 30.0+ kg/m^2^). Time since baseline was the underlying time variable. Unless otherwise specified, analyses were stratified by year of birth (<1929, single years 1929 to 1950, 1950+), year at baseline (single years, 1999 to 2005), and region of residence (10 regions corresponding to regional cancer registries). Relative risks were adjusted for social deprivation (quintiles, based on the Townsend Deprivation Index [12]), educational qualifications (any, none), adult height (<165, 165-169, >=170 cm), smoking status (never, past, current: <10, 10-19, >=20 cigarettes per day), strenuous exercise (never, once/week, more than once/week), alcohol consumption (0, 1-3, 4-6, 7-14, 15+ drinks/week), age at menarche (<12, 12-14, 15+ years), parity (0, 1, 2, 3+), age at first birth (<24, 24+ years), oral contraceptive use (never, ever), age at menopause (<50, 51-54, 55-59, 60+ years), and first-degree family history of breast cancer (yes, no). Women with missing values for all adjustment variables other than height were assigned to a separate category for that variable (<4% for each variable). Where appropriate, estimated relative risks were mutually adjusted for adiposity at other ages.

For risk factors with more than two categories, results in tables and figures are reported in the form of group-specific confidence intervals (g-s CIs), based on the variance of the log risk for each group [13]. However, where comparisons between specific categories are quoted in the text, conventional confidence intervals are given.

Associations between risk and adiposity at different ages were summarised using log-linear trends in risk with BMI at age 10, age 20 and at baseline (i.e. around age 60). For BMI at age 60, trends were calculated by assigning a mean BMI to each category of self-reported baseline BMI, derived from measurements in a subsample of participants around 6 years later [8]. For early life adiposity, a mean BMI at age 10 was assigned to each category of self-reported body size at age 10, and a mean BMI at age 20 was assigned to each category of self-reported clothes size at age 20, based on information collected at relevant ages (11 and 20 years) in those participants who also took part in the NSHD [7].

Summary trends in postmenopausal breast cancer risk with BMI at age 10 and BMI at age 60 were examined by histology, grade, ER status, joint ER and PR status, and by molecular subtype (luminal A, luminal B, HER2-enriched, basal-like). Summary trends were also compared across subgroups of women defined by time since menopause, reproductive factors, family history of breast cancer and various lifestyle characteristics. Heterogeneity across subgroups was assessed by likelihood ratio tests for addition of an interaction term between each subgroup variable and a body size variable.

## Results

Of 837,985 women who took part in the baseline survey, 53,241 were excluded because they had a prior history of cancer. Of the remaining 784,744 women, 11,956 were excluded because they had missing information on body size at age 10 or 20, and a further 14,967 were excluded because they had missing information on recent height and/or weight. Among the remaining 757,821 women, 415,742 had ever used menopausal hormone therapy and so were excluded from the main analyses, although they were included in certain sensitivity analyses. Characteristics of the 342,079 women who were included in the main analyses are shown in eTable 1. Women who reported being plumper as opposed to leaner than their peers at age 10 were more likely to be current smokers and to have greater BMI at baseline. Women who reported being size 16+ as opposed to size <12 at age 20 tended to be taller, drink less alcohol, and have greater BMI at age 60, but less likely to have used oral contraceptives or take regular strenuous exercise.

There was a modest correlation between body size at age 10 and BMI at around age 60 (Spearman correlation 0.14, p<0.0001) and a moderate correlation between clothes size at age 20 and BMI at around age 60 (Spearman correlation 0.34, p<0.0001). The Spearman correlation between body size at 10 and clothes size at 20 was 0.37 (p<0.0001).

**Fig. 1:**
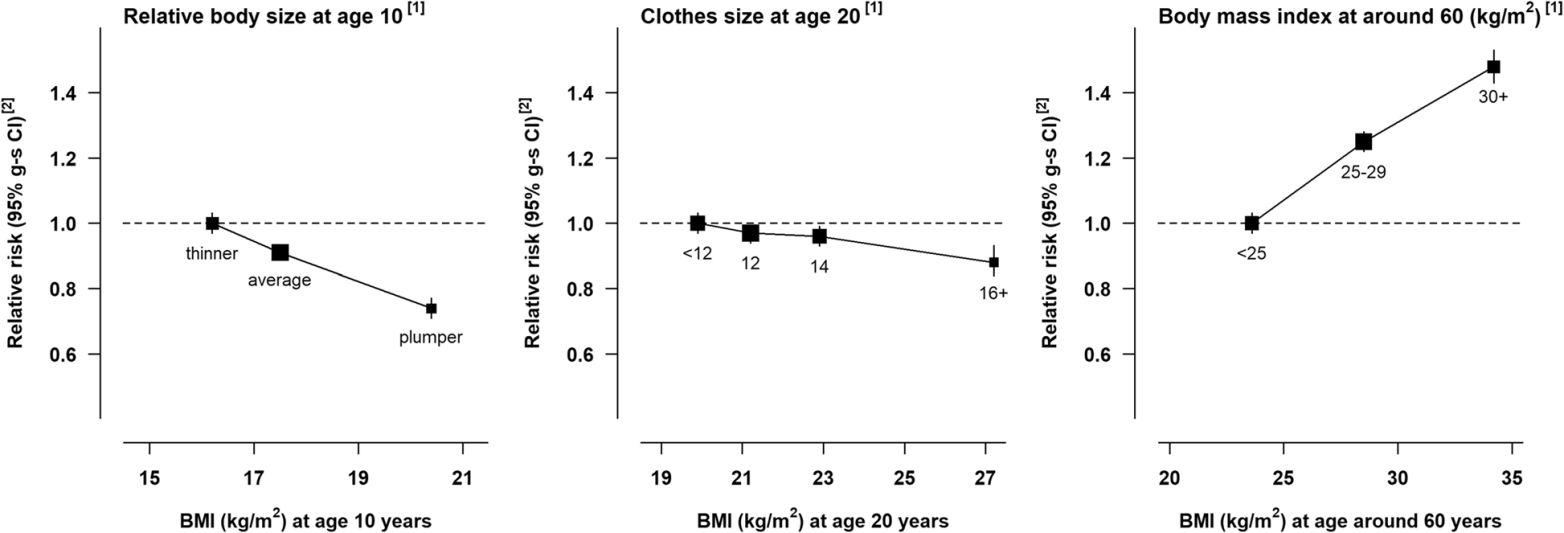
Relative risk of postmenopausal breast cancer by categories of body size in early life and midlife. [1] Relative risks associated with categories of self-reported body size at age 10 and clothes size at age 20 are plotted against mean BMI measures at relevant ages in a subsample of participants who were also included in the National Survey of Health and Development cohort. Relative risks associated with categories of self-reported BMI at around age 60 are plotted against mean measured BMI in a subsample of participants around 6 years later. [2] Relative risks were stratified by year of birth, year at baseline, and region, and adjusted for social deprivation, education, adult height, first-degree family history of breast cancer, smoking, exercise, alcohol consumption, age at menarche, parity and age at first birth, use of oral contraceptives, and age at menopause. Confidence intervals are represented as group-specific confidence intervals (g-s CIs, see Methods); analyses were restricted to never users of menopausal hormone therapy.

During an average follow-up of 14 years, there were 15,506 incident breast cancers. After adjustment for 15 potential confounders, but not for body size at other ages, greater BMI at around age 60 was associated with increased risk of postmenopausal breast cancer, but greater body size at age 10 and, to a lesser extent, clothes size at age 20, were associated with decreased risk (Fig. 1; eTable 2). The estimated RRs per 5kg/m^2^ increase in BMI at age 10, age 20, and at age 60 were 0.70 (0.66-0.74), 0.92 (0.89-0.96) and 1.20 (1.18-1.22), respectively.

**Fig. 2:**
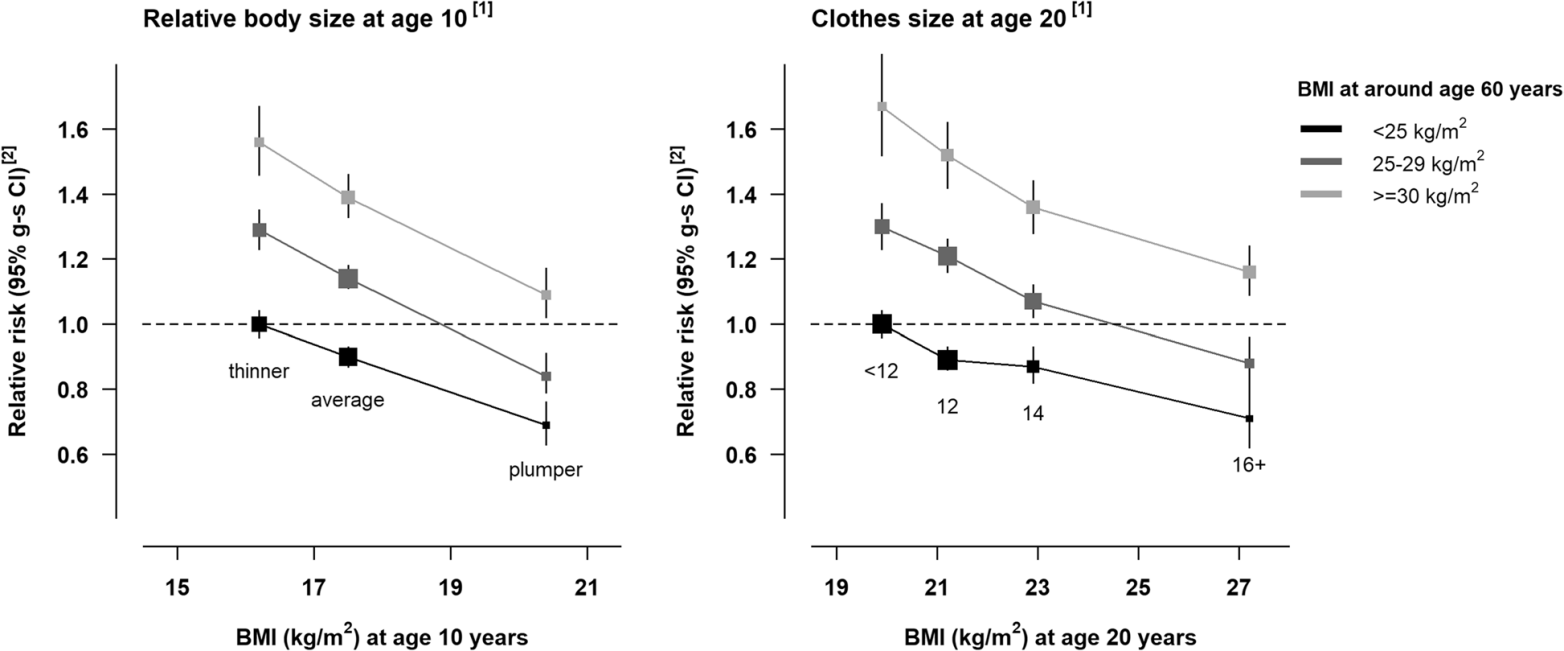
Relative risk of postmenopausal breast cancer by categories of early life body size and BMI at around age 60 years. [1] Relative risks associated with categories of self-reported body size at age 10 and clothes size at age 20 are plotted against mean BMI measures at relevant ages in a subsample of participants who were also included in the National Survey of Health and Development cohort. [2] Relative risks were stratified by year of birth, year at baseline, and region, and adjusted for social deprivation, education, adult height, first-degree family history of breast cancer, smoking, exercise, alcohol consumption, age at menarche, parity and age at first birth, use of oral contraceptives, and age at menopause. Confidence intervals are represented as group-specific confidence intervals (g-s CIs, see Methods); analyses were restricted to never users of menopausal hormone therapy.

The inverse associations with risk of both adiposity at age 10 and at age 20 were consistent across categories of BMI at age 60 (Fig. 2) (p-value for interaction>0.9 in each analysis). After adjustment for BMI at age 60, the estimated RRs per 5kg/m^2^ increase in BMI at age 10 and at age 20 were 0.63 (0.60-0.68) and 0.78 (0.75-0.81), respectively (eTable 2).

The association of BMI at age 60 with risk was considerably attenuated in ever compared with never MHT users (eTable 3) but associations with BMI at age 10 and at age 20 were very similar in ever and never MHT users.

The trend in breast cancer risk with BMI at age 20 (adjusted for BMI at age 60) was attenuated from 0.78 (0.75-0.81) to 0.85 (0.82-0.89) per 5kg/m^2^ after further adjustment for body size at age 10: the corresponding trend with body size at age 10 (adjusted for BMI at age 60) was also attenuated, from 0.63 (0.60-0.68) to 0.69 (0.65-0.74), after adjustment for clothes size at age 20 (results not shown). Subsequent analyses, aimed at contrasting the relationships between risk and early versus midlife BMI, focussed on the strongest opposite associations of body size at age 10 and BMI at age 60.

**Fig. 3:**
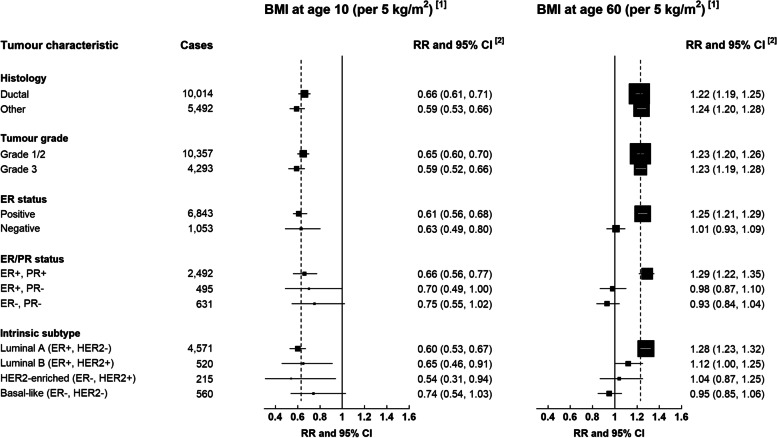
Estimated log-linear trends in breast cancer risk with BMI at age 10 and at around age 60 years, by tumour characteristics. [1] To estimate log-linear trends a mean BMI at around age 10 was assigned to each category of self-reported body size at age 10 based on information collected at age 11 in the subsample of participants who were included in the National Survey of Health and Development cohort. In the case of BMI at around age 60, trends in risk were estimated by assigning a mean BMI to each category of self-reported baseline BMI, derived from measurements taken in a subsample of participants around 6 years later. [2] Relative risks (RRs) and 95% confidence intervals (95%CIs) were stratified by year of birth, year at baseline, and region, and adjusted for social deprivation, education, adult height, first-degree family history of breast cancer, smoking, exercise, alcohol consumption, age at menarche, parity and age at first birth, use of oral contraceptives, age at menopause, and mutually for body mass index at age 10 and around 60 years; analyses were restricted to never users of menopausal hormone therapy.

In these data, information on ER status was available for 7896 breast cancer cases, of which 3654 also had information on PR status, and 5866 also had information on HER2 status. When breast cancer risk was examined according to body size at age 10 and BMI at age 60, after mutual adjustment for each other, the positive association with BMI at age 60 was largely confined to ER+ cancers (p-value for heterogeneity by ER status=0.0000006) (Fig. 3). Further examination of this association by combined ER and PR status showed that the excess risk associated with BMI at age 60 was observed only in those cancers which were PR+ as well as ER+ (p<0.0001 for heterogeneity by joint ER/PR status). The positive association of BMI at age 60 with risk was evident for both the luminal A (RR=1.28, 1.23-1.32) and luminal B subtype (RR=1.12, 1.00-1.25) per 5kg/m^2^ but not for HER2-enriched or basal-like cancers (p<0.0001 for heterogeneity by molecular subtype). There was no evidence of any variation in the association of BMI at age 60 with risk by histology or grade (Fig. 3).

**Fig. 4:**
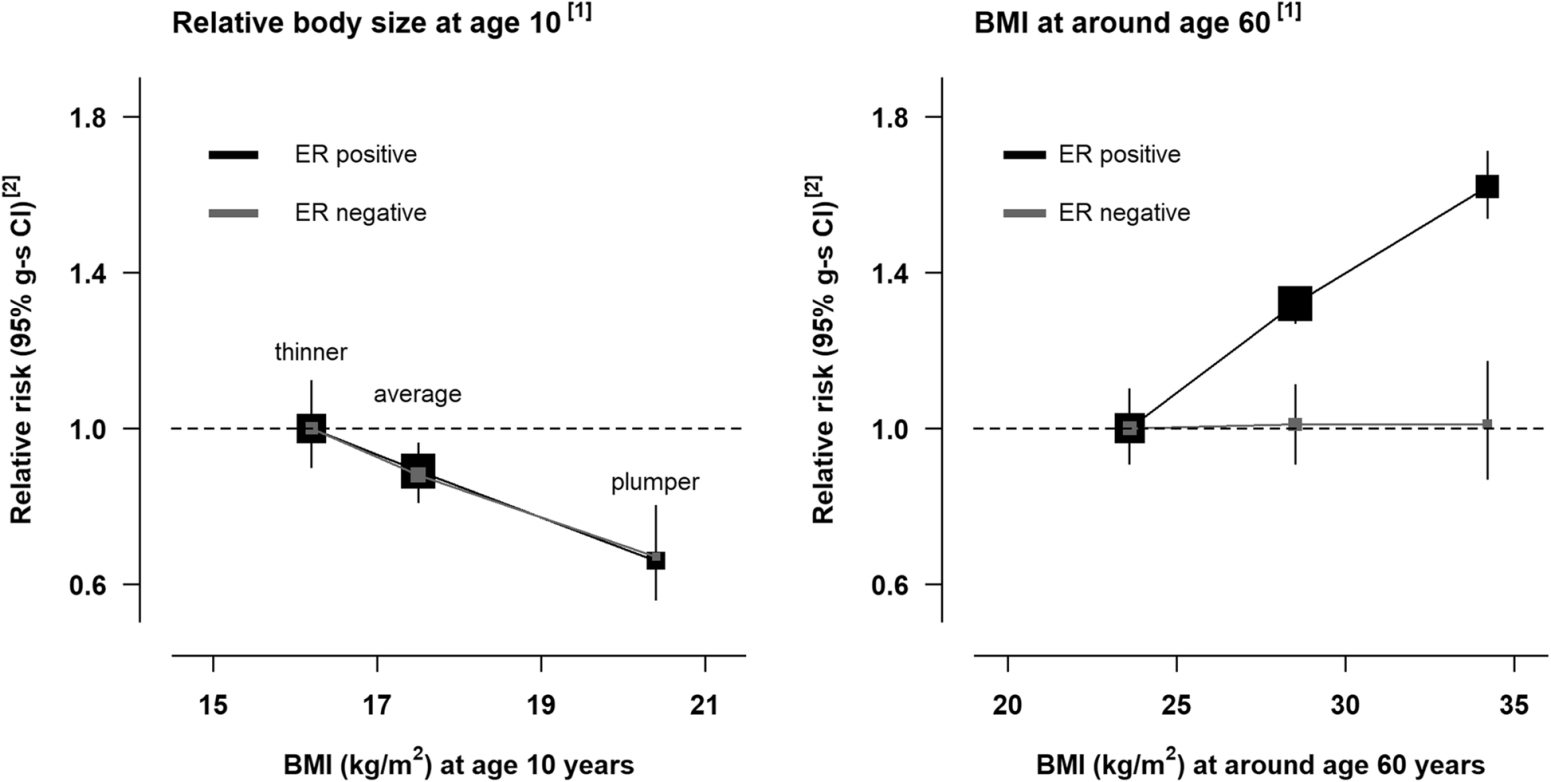
Relative risk of ER positive and ER negative postmenopausal breast cancer by relative body size at age 10 years and body mass index (BMI) at around 60 years among women who had never used menopausal hormone therapy. [1] Relative risks associated with categories of self-reported body size at age 10 are plotted against mean BMI measured at age 11 years in a subsample of participants who were also included in the National Survey of Health and Development cohort. Relative risks associated with categories of self-reported BMI at around age 60 are plotted against mean BMI measured in the subsample of participants around 6 years later. [2] Relative risks were stratified by year of birth, year of baseline, and region, and adjusted for social deprivation, education, adult height, first-degree family history of breast cancer, smoking, exercise, alcohol consumption, age at menarche, parity and age at first birth, use of oral contraceptives, age at menopause, and mutually for body size at age 10 and around age 60 years. Confidence intervals are represented as group-specific confidence intervals (g-s CIs, see Methods); analyses were restricted to never users of menopausal hormone therapy.

The association of BMI at age 10 with breast cancer risk, adjusted for BMI at age 60, was very similar for ER+ (RR=0.61, 0.56-0.68) and ER- cancers (RR=0.63, 0.49-0.80) per 5kg/m^2^ (Fig. 3). Associations with clothes size at age 20, adjusted for BMI at age 60, were also similar for ER+ and ER- cancers (RRs per 5 kg/m^2^: 0.79 (0.74-0.84), 0.79 (0.67-0.93), respectively; results not shown), albeit less marked than those for body size at age 10. Categorical relative risks according to body size at age 10, and BMI at age 60, are shown according to ER status in Fig. 4. When the associations with BMI at age 10 were further examined by joint ER and PR status, there was little evidence of any variation in risk by either ER status or PR status. BMI at age 10 was also associated with a similar reduction in risk of all four molecular subtypes (p-value for heterogeneity=0.6) (Fig. 3).

When trends in risk with BMI at age 10 and at age 60 were examined according to 11 personal characteristics (eFigure 1), there was no strong evidence of variation by any characteristic. There was some evidence of a lesser reduction in risk with greater body size at age 10 in those with a family history of breast cancer. Given the large number of comparisons made this finding was not remarkable, and could reflect a lesser proportionate decrease in risk among women with a family history of breast cancer, who have a greater background risk of breast cancer.

## Discussion

In this prospective study of 342,079 postmenopausal women, including 15,506 breast cancers, women with greater early life body size had a lower risk of both ER+ and ER- cancer subtypes. Although body size in childhood and young adulthood were both associated with a decreased risk of breast cancer, childhood adiposity appeared to be a bigger determinant of any long-term reduction in risk.

Several prospective studies have reported a lower risk of postmenopausal breast cancer among women with greater body size during childhood [2–5]. The largest of these used a 9-level figure drawing for body size at age 5 and 10 years reported during adulthood, and found a reduction in postmenopausal breast cancer risk of 9% per unit increase in perceived body fatness [3]. The only previous study based on measured BMI [5] reported a relative risk after age 50 of 0.73 (0.66–0.86) per 5 unit increase in BMI at age 14, without adjustment for current BMI, similar to the estimate observed in our data for BMI at age 10 (0.70, 0.66-0.74).

Our finding of a significantly lower risk of postmenopausal breast cancer among women with greater body size at age 20 is supported by some [3, 4, 14] but not all [15, 16] previous prospective reports. While it is difficult to disentangle the effects of correlated measures of early life adiposity, our findings suggest that body size in childhood, rather than early adulthood, is the biggest determinant of any long-term reduction in risk.

Midlife adiposity has been shown to primarily affect ER+ breast cancer [1], although its relationship with more refined molecular subtypes is less clear [17]. In contrast, there is little reliable evidence regarding early life adiposity and breast cancer subtypes. To date, three prospective studies have reported on childhood body size and postmenopausal breast cancer risk by ER status: the largest of these found that average BMI during adolescence was associated with a slightly greater reduction in risk of ER- cancer compared with ER-positive cancer [18] but the other two studies found no significant difference by ER status, although they were limited in terms of power [2, 4]. The only previous study to examine the association of early life body size with risk of specific molecular subtypes found that greater childhood adiposity was associated with a reduced risk of all four intrinsic subtypes (18). Findings from studies of body size in young adulthood in relation to breast cancer subtypes have generally been inconclusive, although one reported a significant reduction in postmenopausal breast cancer risk associated with greater average adolescent body fatness (ages 10-20) for both ER+ and ER- disease [3]. To our knowledge, our study is the largest study to date of early life body size in relation to specific subtypes of postmenopausal breast cancer and its findings provide strong evidence that, unlike midlife BMI, adiposity in early life, and particularly in childhood, is associated with a similar substantial reduction in the risk of ER+ and ER- cancers, and in the risk of each of the four main molecular subtypes.

The established associations between adult adiposity and pre- and postmenopausal breast cancer are qualitatively different, but both could be mediated by sex hormones. In postmenopausal women, increased adiposity leads to increased oestrogen synthesis and reduced circulating sex-hormone binding globulin, leading to higher levels of bioavailable oestradiol, which is positively associated with breast cancer risk [19]. In premenopausal women, increased adiposity is thought to disrupt the normal menstrual cycle and consequently lower exposure to oestrogen and progesterone [20] although other mechanisms may also be involved. Our findings support these hypothesised mechanisms in that they show that associations with adult adiposity are largely confined to ER+ disease. PR positivity is generally thought to indicate a functioning oestrogen receptor system, and hence a more hormone-dependent cancer [21], and so our finding that midlife BMI appears to primarily increase the risk of cancers that are both ER+ and PR+ could be viewed as supportive of the hypothesis that the effect of midlife BMI is mediated by hormones.

In contrast, the explanation for the association between early life body size and breast cancer risk is unclear. Greater early life body size is associated with greater adiposity throughout the adult premenopausal years, which, in turn, is associated with reduced risk of premenopausal breast cancer, which may not be reversed until some years after menopause. However, this is unlikely to explain the strong inverse association observed between body size at age 10 and postmenopausal risk given that the average age at diagnosis in these data was 67, and that the association remained after adjustment for clothes size at age 20. Our findings provide convincing new evidence that childhood adiposity has similar associations with ER- and ER+ subtypes, which suggests that it may influence risk through different mechanisms from those that underlie the association with adult adiposity, for example through its effect on growth hormone profiles [22] or breast tissue development [23].

Two large Mendelian randomisation studies have shown that genetically determined adiposity is associated with a substantial reduction in the long-term risk of postmenopausal breast cancer, and that this risk reduction is similar for both ER+ and ER- breast cancer [24, 25]. Given the well-established positive association of adult BMI and postmenopausal breast cancer risk with ER+ disease based on anthropometric measures, these findings suggest that the genetic component of BMI, as captured by polygenic risk scores, is more predictive of early life, rather than midlife, adiposity.

The main strengths of this study are the availability of large-scale prospective data on early life body size, and complete, long-term follow-up for breast cancer incidence. Although tumour characteristic information was not complete in these data, the large sample size allowed for reliable assessment of risks by several key tumour characteristics, including ER status and molecular subtype. Restriction of primary analyses to never MHT users eliminated the potential for MHT use to distort the findings, although sensitivity analysis in MHT users showed similar effects of early life body size. While self-reported early life body size in these data corresponds well with BMI measures taken at relevant ages [7] some misclassification is still possible due to recall bias or other factors. We attempted to minimise the effects of such misclassification by correcting estimated trends in risk across self-reported categories of BMI at a given age using median measures recorded at the relevant age in the subset of women who also took part in the NSHD study. Since the vast majority of the cohort were postmenopausal at recruitment, we were unable to investigate the role of early life body size in relation to premenopausal breast cancer risk.

## Conclusion

Women with greater adiposity in early life, and particularly in childhood, have a lower risk of postmenopausal breast cancer. This apparent protection is evident for both ER+ and ER- breast cancer subtypes suggesting that early life adiposity may affect risk through pathways that are not directly related to sex hormones.

## Supplementary Information

**Additional file 1.**

**Additional file 2.**

**Additional file 3.**

**Additional file 4.**

## Data Availability

Anonymised data used here are available to any qualified researcher upon request to the investigators (addressed to mws.access@ndph.ox.ac.uk ) and to the providers of follow-up data (eg, NHS Digital). The Million Women Study Data Access Policy can be viewed online.

## Abbreviations

BMI: Body Mass Index
RRs: Relative Risks
ER: Oestrogen receptor
PR: Progesterone receptor
HER2: Human epidermal growth factor receptor 2
NSHD: National Survey of Health and Development
MHT: Menopausal Hormone Therapy
