# Supplementary figures and images for: Body size in early life and the risk of postmenopausal breast cancer
Source: BMC Cancer. 2022 Mar 8;22:232. doi: 10.1186/s12885-022-09233-9 (PMC8902765; doi:10.1186/s12885-022-09233-9)

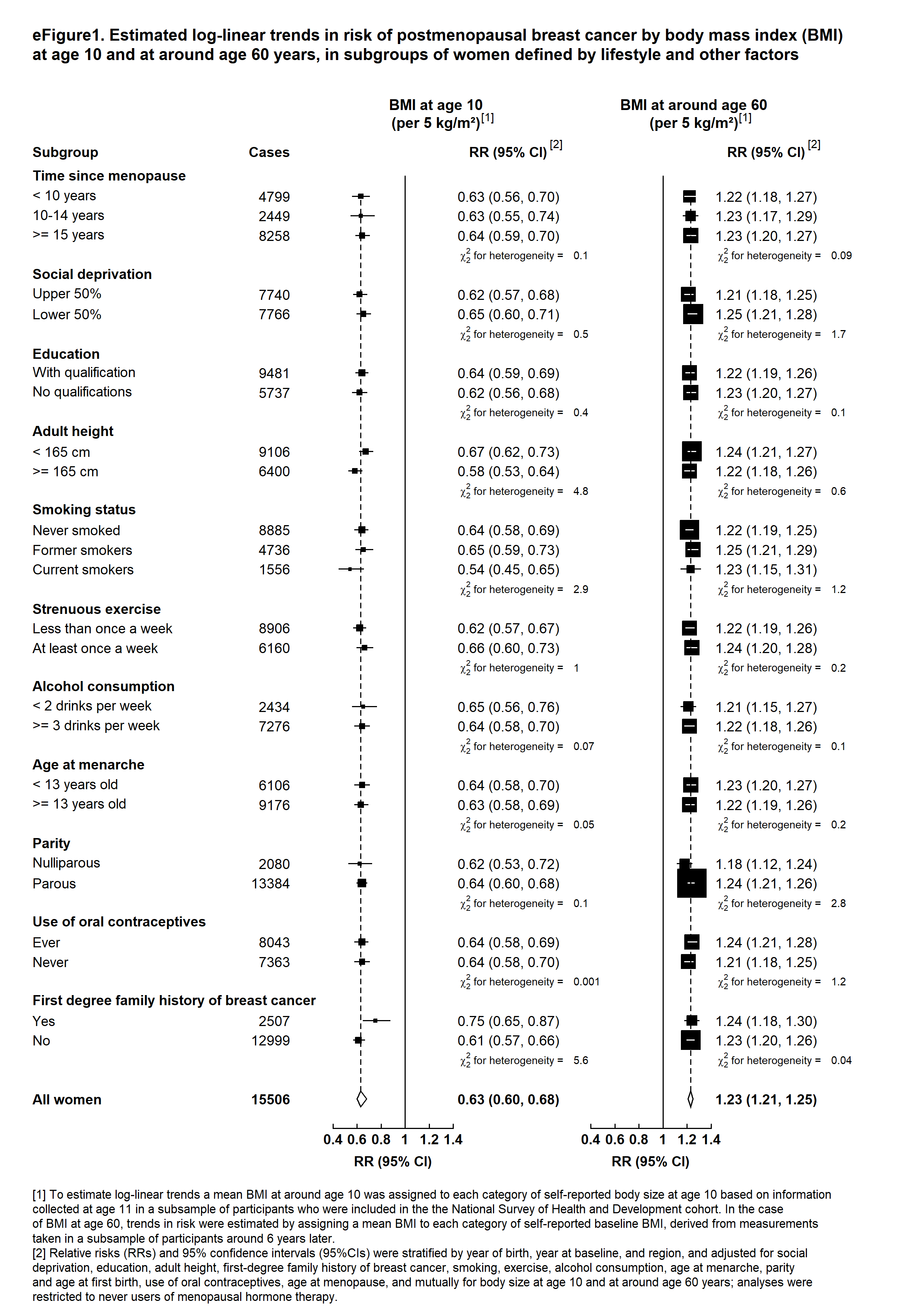

Supplement: Supplementary file 1 — Additional file 1. [file 12885_2022_9233_MOESM1_ESM.tiff]
