# Supplementary material for: Body size in early life and the risk of postmenopausal breast cancer
Source: BMC Cancer. 2022 Mar 8;22:232. doi: 10.1186/s12885-022-09233-9 (PMC8902765; doi:10.1186/s12885-022-09233-9)
Supplement: Supplementary file 2 — Additional file 2. [file 12885_2022_9233_MOESM2_ESM.pdf]

eTable 1. Participant characteristics by body size at age 10 years and clothes size at age 20 years [1]

	Relative body size at age 10 years			Clothes size at age 20 years				All women
	thinner	average	plumper	<12	12	14	16+	
Number of participants	99156	188431	54492	90026	130095	80859	41099	342079
Year of birth (mean and SD)	1940.8 (5.2)	1941.0 (5.3)	1941.9 (5.1)	1941.7 (5.1)	1941.0 (5.2)	1940.6 (5.3)	1940.7 (5.2)	1941.0 (5.2)
Age at start of follow up (mean and SD)	61.0 (5.1)	60.8 (5.1)	59.8 (4.9)	60.1 (5.0)	60.7 (5.1)	61.1 (5.2)	61.0 (5.1)	60.7 (5.1)
**Growth and reproductive factors**								
Adult height (cm, mean and SD)	162.6 (6.8)	162.2 (6.4)	162.0 (6.6)	159.8 (6.0)	162.2 (6.2)	163.9 (6.5)	164.6 (7.0)	162.3 (6.6)
Age at menarche (mean and SD)	13.3 (1.6)	12.9 (1.5)	12.5 (1.6)	13.2 (1.6)	13.0 (1.5)	12.8 (1.5)	12.6 (1.6)	13.0 (1.6)
Any educational qualification (% and n)	57% (55765)	62% (114999)	62% (33421)	63% (55308)	62% (79637)	60% (47766)	53% (21474)	61% (204185)
Parity (mean and SD)	2.2 (1.3)	2.1 (1.2)	2.0 (1.3)	2.1 (1.2)	2.1 (1.2)	2.1 (1.3)	2.0 (1.3)	2.1 (1.2)
Age at first birth if parous (mean and SD)	24.4 (4.3)	24.5 (4.2)	24.5 (4.4)	24.5 (4.3)	24.4 (4.2)	24.5 (4.3)	24.3 (4.4)	24.4 (4.3)
Age at menopause (mean and SD)	49.3 (4.9)	49.5 (4.7)	49.4 (4.9)	49.4 (4.7)	49.5 (4.7)	49.5 (4.8)	49.3 (5.2)	49.4 (4.8)
**Lifestyle factors at study baseline**								
Highest fifth of social deprivation (% and n)	22% (21734)	19% (36098)	22% (11785)	20% (17980)	19% (24915)	20% (16246)	25% (10476)	20% (69617)
Body mass index at baseline (kg/m^2, mean and SD)	25.8 (4.5)	26.2 (4.5)	28.6 (5.9)	24.5 (3.8)	25.7 (4.0)	27.5 (4.7)	31.1 (6.2)	26.4 (4.9)
Ever used oral contraceptives (% and n)	53% (52566)	52% (98175)	55% (29545)	58% (51525)	54% (69329)	51% (40723)	46% (18709)	53% (180286)
Current smoker (% and n)	9% (9277)	11% (20191)	13% (7223)	11% (10030)	10% (13582)	10% (8415)	11% (4664)	11% (36691)
Strenuous exercise at least once a week (% and n)	40% (38870)	43% (78555)	40% (21201)	44% (38111)	43% (54809)	41% (31995)	34% (13711)	42% (138626)
Alcohol consumption of 7+ drinks per week (% and n)	23% (23232)	24% (45894)	24% (12926)	27% (24277)	25% (33147)	22% (18046)	16% (6582)	24% (82052)
**Follow-up**								
Person-years (mean and SD)	14.1 (3.9)	14.1 (3.8)	14.0 (3.9)	14.2 (3.7)	14.2 (3.8)	14.1 (3.9)	13.7 (4.2)	14.1 (3.8)
Invasive breast cancer cases	4887	8569	2050	4122	5907	3728	1749	15506

[1] Analyses were restricted to never users of menopausal hormone therapy.
