# Supplementary material for: Body size in early life and the risk of postmenopausal breast cancer
Source: BMC Cancer. 2022 Mar 8;22:232. doi: 10.1186/s12885-022-09233-9 (PMC8902765; doi:10.1186/s12885-022-09233-9)
Supplement: Supplementary file 4 — Additional file 4. [file 12885_2022_9233_MOESM4_ESM.pdf]

eTable 3. Relative risk (RR) of postmenopausal breast cancer by BMI at baseline and body size at age 10 and age 20 years, among women who had ever used menopausal hormone therapy

	Mean BMI [1]	cases	Stratified for year of birth, year at baseline, and region, and adjusted for social deprivation, education, and height (RR, 95% g-sCI)	Additionally adjusted for 9 other factors [2] (RR, 95% g-sCI)	Further adjusted for BMI at around age 60 years (RR, 95% g-sCI)
**BMI at study baseline (around age 60 years)**					
less than 25.0	23.6	9695	1.00 (0.98-1.02)	1.00 (0.98-1.02)	
25.0-29.9	28.1	8720	1.12 (1.10-1.15)	1.13 (1.11-1.15)	
30.0 or greater	34.7	3871	1.18 (1.15-1.22)	1.21 (1.17-1.25)	
			p(het)<0.0001	p(het)<0.0001	
**Trend (per 5 kg/m^2)**		**22286**	**1.09 (1.07-1.10)**	**1.10 (1.08-1.12)**	
**Relative body size at age 10 years**					
Thinner	16.2	7990	1.00 (0.98-1.02)	1.00 (0.98-1.02)	1.00 (0.98-1.02)
Average	17.5	11569	0.92 (0.90-0.93)	0.90 (0.89-0.92)	0.89 (0.88-0.91)
Plumper	20.4	2727	0.76 (0.73-0.79)	0.73 (0.70-0.76)	0.70 (0.68-0.73)
			p(het)<0.0001	p(het)<0.0001	p(het)<0.0001
**Trend (per 5 kg/m^2)**		**22286**	**0.72 (0.69-0.76)**	**0.69 (0.66-0.73)**	**0.66 (0.63-0.70)**
**Clothes size at age 20 years**					
<12	19.9	7289	1.00 (0.98-1.02)	1.00 (0.98-1.02)	1.00 (0.97-1.03)
12	21.2	8769	0.98 (0.96-1.00)	0.98 (0.96-1.00)	0.95 (0.93-0.97)
14	22.9	4535	0.93 (0.90-0.96)	0.93 (0.90-0.95)	0.86 (0.83-0.88)
16+	27.2	1693	0.84 (0.80-0.88)	0.82 (0.79-0.87)	0.73 (0.69-0.76)
			p(het)<0.0001	p(het)<0.0001	p(het)<0.0001
**Trend (per 5 kg/m^2)**		**22286**	**0.88 (0.85-0.92)**	**0.87 (0.84-0.91)**	**0.80 (0.77-0.83)**

[1] Mean BMI within self-reported body size categories was based on measured BMI in a subsample of 4000 Million Women Study participants on average 6 years after report; mean BMI within categories of body size at age 10 and clothes size at age 20 was based on BMI measured or reported at relevant ages in the subset of participants included in the National Survey of Health and Development Study.

[2] Smoking, exercise, alcohol consumption, age at menarche, parity and age at first birth, use of oral contraceptives, age at menopause, and first-degree family history of breast cancer.
